# Magnitude of problematic anger and its predictors in the Millennium Cohort

**DOI:** 10.1186/s12889-020-09206-2

**Published:** 2020-07-27

**Authors:** Amy B. Adler, Cynthia A. LeardMann, Kimberly A. Roenfeldt, Isabel G. Jacobson, David Forbes, for the Millennium Cohort Study Team

**Affiliations:** 1 Walter Reed Army Institute of Research, Silver Spring, MD USA; 2 Deployment Health Research Department, Naval Health Research Center, 140 Sylvester Road, San Diego, CA 92106 USA; 3 Leidos, 11951 Freedom Drive, Reston, VA USA; 4 Phoenix Australia - Centre for Posttraumatic Mental Health, Department of Psychiatry, University of Melbourne, Melbourne, Australia

**Keywords:** Anger, Cohort studies, Military

## Abstract

**Background:**

Problematic anger is intense anger associated with elevated generalized distress and that interferes with functioning. It also confers a heightened risk for the development of mental health problems. In military personnel and veterans, previous studies examining problematic anger have been constrained by sample size, cross-sectional data, and measurement limitations.

**Methods:**

The current study used Millennium Cohort survey data (*N* = 90,266) from two time points (2013 and 2016 surveys) to assess the association of baseline demographics, military factors, mental health, positive perspective, and self-mastery, with subsequent problematic anger.

**Results:**

Overall, 17.3% of respondents reported problematic anger. In the fully adjusted logistic regression model, greater risk of problematic anger was predicted by certain demographic characteristics as well as childhood trauma and financial problems. Service members who were in the Army or Marines, active duty (vs. reserves/national guard), and previously deployed with high levels of combat had increased risk for problematic anger. Veterans were also more likely to report problematic anger than currently serving personnel. Mental health predictors included posttraumatic stress disorder (PTSD), major depressive disorder (MDD), and comorbid PTSD/MDD. Higher levels of positive perspective and self-mastery were associated with decreased risk of problematic anger.

**Conclusion:**

Not only did 1 in 6 respondents report problematic anger, but risk factors were significant even after adjusting for PTSD and MDD, suggesting that problematic anger is more than an expression of these mental health problems. Results identify potential targets of early intervention and clinical treatment for addressing problematic anger in the military and veteran context.

## Background

Anger, like any other emotion, is a normal human experience. In certain contexts, and when expressed appropriately, it may be useful. In an occupational context, anger may be an accepted emotion [1] and associated with benefits such as prosocial advocacy, promoting critical change, or improving subordinate performance [2]. In the military, anger may be culturally acceptable, and many service members report that they believe anger is helpful to them in functioning, although high levels of anger have been associated with worse outcomes [3]. Depending on its frequency, intensity, and duration, anger can be considered “problematic anger” when it reaches a point where it is associated with elevated generalized distress, and begins to interfere with functioning [4]. Problematic anger may also impede interpersonal relationships particularly when expressed outwardly towards others [4]. For military personnel, problematic anger may be a gateway to major disruption, both personally and professionally. In military and veteran personnel, problematic anger has been associated with mental health conditions, including posttraumatic stress disorder (PTSD [3, 5];), unhealthy habits and unnecessary risk taking [6], as well as relationship dysfunction [7]. Problematic anger and aggression are also risk factors for intent to harm others [5], interpersonal violence [8] and suicide-related outcomes [9]. While there is evidence that problematic anger is a significant concern in the civilian community [10], it is particularly important to understand the extent of problematic anger in military personnel and veterans and the key factors that contribute to its development and expression. The importance of this issue is heightened given the elevated risk of mental health difficulties in military personnel and veterans compared to civilians [11] and indications in meta-analyses that anger in veterans with PTSD is greater than that in civilians with PTSD [12].

Anger may be particularly relevant to the military in several ways. Not only does more problematic anger appear to be associated with combat exposure over time [6, 13], but anger frequently persists over the course of the post-deployment period [13], negatively impacting service members’ ability to adjust following deployment and increasing their risk of developing PTSD [14, 15]. Besides this increased risk, problematic anger has important implications for clinical treatment, including that evidence-based treatment of PTSD may be less effective for veterans reporting high levels of co-occurring anger [16]. In addition, anger weakens PTSD treatment response in military veterans whether they have deployed to combat or peacekeeping missions [17]. In addition, anger has negative implications for job performance; anger-related reactions have been associated with unethical behavior during combat [18], alcohol problems [14], poor decision making [19], and blaming and retaliation [19]. Finally, it is important to consider the degree to which anger is a relatively acceptable emotion in the culture of the military [3], and thus may be reinforced in that occupational context.

Despite the evidence documenting the impact that problematic anger may have on individual health, relationships, and occupational functioning, the previous studies have been limited by small samples and cross-sectional designs. Importantly, one cross-sectional study in the UK demonstrated the importance of demographic and mental health problems in estimating high anger scores in a large military sample [14]. The present study builds on these findings in several ways. First, the present study examined a large, diverse and comprehensive sample that includes veterans. Second, we introduced other risk factors such as history of sexual assault, combat severity, and financial problems along with potential mitigating variables. Third, by examining data collected at two points in time, we were able to assess the independent contribution of mental health problems reported at baseline in the modeling of problematic anger reported several years later. Finally, the present study also used a validated measure of anger that allows for an estimate of prevalence.

While the Spielberger State - Trait Anger Expression Inventory-2 (STAXI-2 [20]) is considered the gold standard for measuring anger, it is often too long for inclusion in studies designed to target a wide range of concerns. Therefore, the present study relied on the five-item Dimension of Anger Reactions scale (DAR-5 [21, 22]; that allowed for an estimate of prevalence of problematic anger. The DAR-5 has been validated against the STAXI-2 [20] and has an established cut-off to identify problematic anger in both military and civilian populations [21, 23].

The present study leveraged data from the Department of Defense’s largest and longest ongoing prospective study of service members and veterans, the Millennium Cohort Study [24]. This first investigation of anger in the Millennium Cohort Study provides a unique opportunity to describe the magnitude of problematic anger in a large sample of service members and veterans, and to determine which individual and occupational factors are associated with problematic anger.

## Methods

### Study population

Established in 2001, the Millennium Cohort Study is a prospective cohort study investigating the long-term health effects related to military service both during service and after separation from the military [24]. The sample for the Cohort was randomly selected from US military rosters, with over-sampling of selected subgroups of interest, as previously described [25, 26]. Service members were enrolled into four panels between 2001 and 2013, resulting in a total of 201,619 participants across all service branches, including both active duty and Reserves and National Guard (Reserve/Guard) members (27.3% cumulative baseline response rate). Previous studies have found the Millennium Cohort to be a representative sample of service members in terms of health status, and analyses on weighting for nonresponse have not identified changes in metrics for mental disorders [27, 28]. Furthermore, using data from the Millennium Cohort, only slight differences between weighted means and nonweighted means for numerous mental and physical health conditions have been found; therefore, nonweighted data were used for the current study [29, 30].

After enrollment, participants were requested to complete follow-up surveys that are accessed online or via postal mail approximately every three to five years, even after they leave military service. These surveys collected information about behavioral, physical, and mental health, as well as service-related experiences. A detailed description of the Millennium Cohort has been previously published [24].

### Inclusion criteria

Cohort members who completed both the 2011–2013 and 2014–2016 surveys, referred to as “baseline” and “follow-up” respectively for this study, were eligible (*n* = 92,614) for the current study. Of these participants, those who were missing 3 or more of the items (*n* = 2348) from the DAR-5 [21, 22] at follow-up were excluded from this study (analytic sample *N* = 90,266, 97.5% of the eligible sample).

### Measures

#### Problematic anger

Problematic anger was assessed at follow-up from the DAR-5, a validated instrument introduced on the follow-up survey (Cronbach’s alpha = 0.91). Participants responded to the question “Indicate the degree to which each statement describes your feelings or behavior:” with a 5-point Likert scale ranging from 1 (*Not at all*) to 5 (*Very Much*) for each of the five items (e.g., when I get angry, I get really mad; when I get angry at someone, I want to hit or clobber the person). Responses were summed and problematic anger (no/yes) was determined using the established cut-off of 12 points or higher [22]. The DAR-5 measure [21] has demonstrated robust convergent, concurrent and discriminant validity. In addition, the recommended cut off, aligned with the 75th percentile of the STAXI-2 [20], has been associated with psychological distress and functional impairment [22, 23] and utilized in numerous studies [31–33].

#### Predictors of anger

##### Demographics and military experiences

Marital status and educational attainment were assessed at baseline using self-reported survey data. The other demographic and military characteristics were obtained at baseline from the Defense Manpower Data Center personnel files including age, sex, race/ethnicity, military service branch, military component, pay grade, and military separation status. Recent deployment experience in support of the operations in Iraq and Afghanistan was defined as occurring between baseline and follow-up. It was assessed using electronic deployment data from Defense Manpower Data Center combined with participants’ responses to 13 self-reported combat experiences (e.g., “being attacked or ambushed”, “receiving small arms fire”) at follow-up. Those without a deployment between baseline and follow-up were classified as not recently deployed. Using methods similar to prior studies [34], individuals with a recent deployment were classified as deployed with no combat (endorsed 0 combat events), or deployed with low combat (endorsed 1–3 combat events), medium combat (endorsed 4–10 combat events), or high combat (endorsed 11–13 combat events). Due to the small number of participants reporting high levels of combat experiences, the two highest categories (medium and high) were combined into one category for this study.

##### Life stressors

Childhood traumatic experiences (e.g. childhood sexual abuse) were asked for the first time of participants at the 2016 (follow-up) survey. These four items from the Juvenile Victim Questionnaire [35] assessed traumatic experiences that occurred prior to age 18, and were summed (0 to 4) [35]. Sexual assault was ascertained at baseline based on positive endorsement to one self-reported item (i.e., suffered forced sexual relations or sexual assault within the last 3 years). Financial problems were ascertained at baseline based on endorsement to one self-reported item (i.e., financial problems or worries within the last 4 weeks).

##### Psychological health/well-being

Probable posttraumatic stress disorder (PTSD) was assessed at baseline using the PTSD Checklist−Civilian Version (PCL-C), used to rate the severity of 17 PTSD symptoms [36]. Based on criteria from the Diagnostic and Statistical Manual of Mental Disorders 4th edition (DSM-IV), participants were classified as having probable PTSD if they reported a moderate or greater level of at least one intrusion symptom, three avoidance symptoms, and two hyperarousal symptoms [37]. Probable major depressive disorder (MDD) was assessed at baseline using eight Patient Health Questionnaire (PHQ) items, which correspond to a depression diagnosis in the DSM-IV [37]. Based on criteria from the DSM-IV, probable MDD was defined as endorsing at least 5 items as “more than half the days” or “nearly every day”, in which one symptom was depressed mood or anhedonia [38]. To assess the joint impact of PTSD and MDD, PTSD and MDD were combined to create a 4-level variable (neither PTSD nor MDD, PTSD only, MDD only, and comorbid PTSD and MDD). Problem drinking was assessed at baseline from endorsements of any of the 5 PHQ alcohol items (e.g., drank while working or taking care of responsibilities, missed or were late for work or other activities because you were drunk or hung over, drove a car after drinking too much) more than once in the last 12 months [39].

Potential mitigating factors for anger were also assessed. A version of the Posttraumatic Growth Inventory-Short Form (PTGI-SF) [40] was included at baseline to measure positive perspective. Unlike the original PTGI-SF, which asks for retrospective assessment of positive changes in personal perception following a traumatic event, the 11-item version used in the present study assessed current perspectives and did not reference a specific trauma. This current standing version was composed of the PTGI-SF [41] and an additional item about compassion for others. Mean scores were calculated based on participant responses to each item (e.g., “I know that I can handle difficulties,” “I have a religious faith,” “I have a sense of closeness with others”) on a 6-point Likert scale from 0 (*Not at all*) to 5 (*To a very great degree*). Self-mastery was ascertained at baseline using 3 items (e.g., I can do just about anything I really set my mind to do) from Pearlin and Schooler’s Self-Mastery Scale [42]. Mean scores were calculated based on responses on a 5-point Likert scale from 1 (*Strongly disagree*) to 5 (*Strongly agree*).

### Statistical analysis

Descriptive analyses and chi-square tests were used to compare demographic, military, life stressors, and psychological health/well-being characteristics by problematic anger status. Bivariate logistic regression analyses were performed to assess the relationship between each factor (e.g., sex, age, marital status, educational attainment, race/ethnicity, military service branch, military service component, military separation status, recent combat severity, childhood traumatic experiences, sexual assault, financial problems or worries, mental health status, positive perspective, and self-mastery) and problematic anger. A multivariable logistic regression model was performed to determine which factors were significantly associated with problematic anger. In order to determine the influence of each factor above and beyond the other variables, all factors were included in the multivariable model regardless of statistical significance. To assess the percent of problematic anger that could be reduced in this population if certain exposures/factors were eliminated, the population attributable risk percent (PAR%) was calculated for each life stressor and psychological/well-being factor [the prevalence of the factor among those with problematic anger multiplied by the adjusted odds ratio (AOR) minus 1 divided by the OR multiplied by 100 (prevalence among cases × [(OR − 1)/OR] × 100%)]. Multicollinearity was assessed using a variance inflation factor of four or higher. *P*-values of less than 0.05 were regarded as statistically significant. All analyses were completed using SAS statistical software, version 9.4 (SAS Institute, Inc., Cary, NC).

#### Missing data

Among the study participants (*n* = 90,266), each DAR-5 item had less than 0.4% missing. All model predictors had less than 3.1% missing with the exception of the childhood traumatic experiences. Each of the four items had approximately 3% missing and an additional 3% who responded “prefer not to answer.” These responses were therefore classified into their own category to maintain these participants in analyses. For all other items, we used multiple imputation to maintain participants. Discriminant functions estimated categorical variables to ensure imputed data were integers within the range of possible values. A total of 50 imputed datasets were generated [43].

## Results

Of the 90,266 study participants, the population was predominantly male (70.6%), 26 to 39 years of age (50.3%), non-Hispanic whites (76.9%), and married (66.5%). The largest proportion of participants had at least some college education (91.4%), served in the Army (44.4%), served on active duty (55.1%), had separated from service by follow-up (62.3%), and had not deployed between baseline and follow-up (88.4%). Baseline characteristics are presented in Table 1. Overall, 15,600 (17.3%) participants had problematic anger at follow-up. Notably, participants with problematic anger, compared to those without, were proportionally more likely to be younger (< 40 years old); less educated (high school or less); separated from the military, and in the Army or Marines (all *p* < 0.001). In addition, participants who experienced any type of life stressor (all *p* < 0.001) or had a probable mental disorder (p < 0.001) or problem drinking (*p* < 0.001) were proportionally more likely to report problematic anger (Table 1). Participants with problematic anger had lower mean scores for both positive perspective (p < 0.001) and self-mastery (p < 0.001) compared to those without problematic anger.

**Table 1:** Frequencies and Percentage of Baseline Characteristics by Problematic Anger Status

Baseline Characteristics*	Study Sample n	No Problematic Anger		Problematic Anger	
		n	%	n	%
Sample	90,226	74,626	82.7	15,600	17.3
**Demographics**					
Sex					
Male	63,650	52,643	70.5	11,007	70.6
Female	26,576	21,983	29.5	4593	29.4
Age					
17–25 years	9682	7438	10.0	2244	14.4
26–39 years	45,395	36,369	48.7	9026	57.9
40 years or more	35,149	30,819	41.3	4330	27.8
Educational attainment					
High school or less	7776	5673	7.6	2103	13.5
Some college/Associate’s degree	41,244	32,394	43.4	8850	56.7
Bachelor’s degree	23,132	20,030	26.8	3102	19.9
Graduate degree	18,071	16,526	22.1	1545	9.9
Race/ethnicity					
White, non-Hispanic	69,361	57,973	77.7	11,388	73.0
Black, non-Hispanic	8693	7059	9.5	1634	10.5
Other	12,122	9549	12.8	2573	16.5
Marital status					
Never married	17,362	14,134	18.9	3228	20.7
Married	59,961	50,415	67.6	9546	61.2
Formerly married	12,903	10,077	13.5	2826	18.1
**Military**					
Service branch					
Army	40,022	31,119	41.7	8903	57.1
Navy/Coast Guard	16,581	14,120	18.9	2461	15.8
Marine Corps	6622	4834	6.5	1788	11.5
Air Force	27,001	24,553	32.9	2448	15.7
Service component					
Reserves/National Guard	40,540	33,897	45.4	6643	42.6
Active duty	49,686	40,729	54.6	8957	57.4
Military status at 2016					
Not separated (currently serving)	33,974	29,755	39.9	4219	27.0
Separated (veteran)	56,252	44,871	60.1	11,381	73.0
Recent deployment experience, 2013–2016 ^a^					
Not recently deployed	79,718	65,203	87.4	14,515	93.0
Deployed, no combat	3178	2982	4.0	196	1.3
Deployed, low combat	3130	2764	3.7	366	2.3
Deployed, medium/high combat	1739	1416	1.9	323	2.1
**Life stressors**					
Childhood traumatic experiences^b^					
None	49,179	43,560	58.4	5619	36.0
1 experience	18,311	14,899	20.0	3412	21.9
2 experiences	11,336	8404	11.3	2932	18.8
3 or more experiences	2275	1394	1.9	881	5.6
Missing/prefer not to answer	9125	6369	8.5	2756	17.7
Sexual Assault					
No	85,908	71,441	95.7	14,467	92.7
Yes	2057	1388	1.9	669	4.3
Financial problems					
Not bothered	49,001	44,009	59.0	4992	32.0
Bothered a little	29,351	23,441	31.4	5910	37.9
Bothered a lot	10,942	6420	8.6	4522	29.0
**Psychological health/well-being**					
Mental health status^c^					
None	79,553	69,699	93.4	9854	63.2
MDD only	1152	671	0.9	481	3.1
PTSD only	4928	2315	3.1	2613	16.8
Comorbid PTSD/MDD	3588	1166	1.6	2422	15.5
Problem drinking^d^					
No	78,065	66,243	88.8	11,822	75.8
Yes	9370	6129	8.2	3241	20.8
Positive Perspective^e^ (Mean ± SD)	3.7 ± 0.9	3.8 ± 0.8		3.1 ± 1.0	
Self-Mastery^f^ (Mean ± SD)	4.0 ± 0.7	4.0 ± 0.7		3.7 ± 0.8	

*Characteristics are assessed at 2013 unless noted otherwise. Some columns (n and %) do not add up to column total due to missing data. Chi-square test of independence between each characteristic and problematic anger was statistically significant (*p* < 0.05), except sex

^a^Based on deployments between baseline and follow-up in support of the operations in Iraq and Afghanistan

^b^Based on 4 items assessed at follow-up: childhood sexual abuse, neglect, verbal abuse, and physical abuse

^c^Mental health status based on probable major depressive disorder (MDD) and/or posttraumatic stress disorder (PTSD) based on Diagnostic and Statistical Manual of Mental Disorders 4th edition (DSM-IV) criteria

^d^Based on endorsing any of the five Patient Health Questionnaire (PHQ) alcohol items

^e^Assessed from the mean score of all 11 items from the Current State -Posttraumatic Growth Index – Short Form (C-PTGI-SF)

^f^Assessed from the mean score of 3 self-mastery items from Pearlin and Schooler’s Self - Mastery Scale

**Fig. 1:**
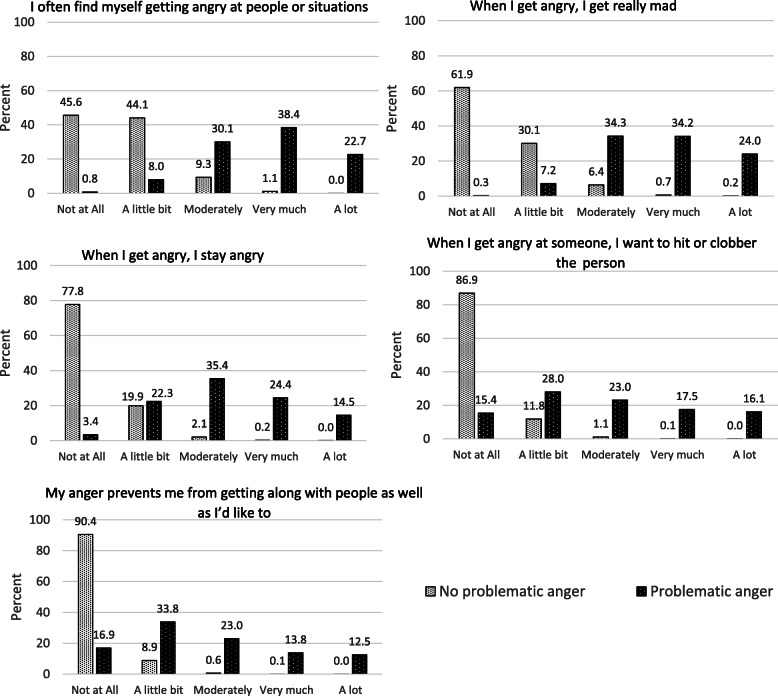
Distribution of responses to Dimensions of Anger Reactions −5 items by problematic anger status

Appreciable endorsement of DAR-5 items was calculated as any response from “moderately” to “a lot”. Across participants, 24.3% endorsed “I often find myself getting angry at people or situations”, 22.0% endorsed “When I get angry, I get really mad”, 14.8% endorsed “When I get angry, I stay angry”, 10.8% endorsed “When I get angry at someone, I want to hit or clobber the person”, and 9.1% endorsed “My anger prevents me from getting along with people as well as I’d like to”. Responses to the DAR-5 items by problematic anger status are presented in Fig. 1. For each item, endorsement was much higher among those with problematic anger.

Table 2 provides ORs for problematic anger at follow-up, after adjusting for all variables in the table. Given that there was no meaningful difference between results with and without multiple imputation, the imputed results are displayed. After adjustment, demographic factors found to be significantly associated with problematic anger included male sex, younger age, less educational attainment, black non-Hispanic and other race/ethnicities (compared with white non-Hispanic), and marital status. During the model building process a significant interaction between marital status and age was discovered (*p* < .01), which indicated that married and formerly married participants were significantly more likely to have problematic anger in the younger age groups (17–39 years old), but that marital status was not associated with problematic anger among the oldest participants (40+ years old). For military factors, Navy/Coast Guard [AOR = 0.65; 95% CI: 0.61, 0.69] and Air Force [AOR = 0.52, 95% CI: 0.49, 0.55] members were significantly less likely than Army personnel to have problematic anger. Active duty service members were significantly more likely to have problematic anger [AOR = 1.28; 95% CI 1.23, 1.34] compared with Reservists and National Guardsmen. Separation from military service was also associated with higher odds of problematic anger [AOR = 1.45; 95% CI 1.38, 1.52], as was deployment with medium/high combat severity between baseline and follow-up [AOR = 1.34; 95% CI 1.16, 1.54]. In a sub-analysis, when combat was categorized as a 3-level variable (not recently deployed, deployed without combat, and deployed with combat), those who experienced any combat had increased odds for problematic anger [AOR = 1.13, 95% CI 1.02, 1.24].

**Table 2:** Adjusted^a^ Odds Ratios of Problematic Anger

Characteristic	AOR (95%CI)
**Demographics**	
Sex (ref: female)	
Male	1.07 (1.02, 1.12)
Age (ref: 40+ years old)	
17–25 years old	1.79 (1.66, 1.93)
26–39 years old	1.69 (1.61, 1.77)
Educational attainment (ref: graduate degree)	
High school or less	1.98 (1.81, 2.15)
Some college or Associate’s degree	1.67 (1.56, 1.78)
Bachelor’s degree	1.26 (1.17, 1.35)
Race/ethnicity (ref: white, non-Hispanic)	
Black, non-Hispanic	1.22 (1.14, 1.31)
Other	1.20 (1.14, 1.27)
Marital status^b^ (ref: never married)	
Married	1.21 (1.14, 1.27)
Formerly married	1.10 (1.03, 1.18)
**Military service**	
Service branch (ref: Army)	
Air Force	0.52 (0.49, 0.55)
Navy/Coast Guard	0.65 (0.61, 0.69)
Marine Corps	1.02 (0.95, 1.09)
Service component (ref: Reserves/National Guard)	
Active duty	1.28 (1.23, 1.34)
Military status at 2016 (ref: not separated/currently serving)	
Separated (veteran)	1.45 (1.38, 1.52)
Recent deployment experience, 2013–2016^c^ (ref: not recently deployed)	
Deployed, no combat	0.57 (0.48, 0.67)
Deployed with low combat	1.00 (0.88, 1.13)
Deployed with medium/high combat	1.34 (1.16, 1.54)
**Life stressors**	
Childhood traumatic experiences^d^ (ref: none)	
1 experience	1.45 (1.38, 1.53)
2 experiences	1.97 (1.86, 2.08)
3 or more experiences	2.70 (2.43, 3.00)
Missing/prefer not to answer	2.11 (1.99, 2.25)
Sexual assault (ref: no)	
Yes	1.09 (0.97, 1.23)
Financial problems (ref: not bothered)	
Bothered a little	1.43 (1.37, 1.50)
Bothered a lot	1.94 (1.82, 2.06)
**Psychological health/well-being**	
Mental health status^e^ (ref: none)	
MDD only	2.12 (1.86, 2.42)
PTSD only	3.52 (3.28, 3.78)
Comorbid PTSD/MDD	4.43 (4.09, 4.80)
Problem drinking (ref: no)^f^	
Yes	1.46 (1.38, 1.55)
Positive Perspective^g^	0.72 (0.70, 0.74)
Self-Mastery^h^	0.84 (0.82, 0.87)

^a^Data are reported from one model that adjusted for all variables in this table

^b^A significant interaction with age and marital status was found, which indicated that marital status was not significantly associated with problematic anger among the 40+ year olds

^c^ Based on deployments between baseline and follow-up in support of the operations in Iraq and Afghanistan

^d^Based on 4 items assessed at follow-up: childhood sexual abuse, neglect, verbal abuse, and physical abuse

^e^Mental health status based on probable major depressive disorder (MDD) and/or posttraumatic stress disorder (PTSD) based on Diagnostic and Statistical Manual of Mental Disorders 4th edition (DSM-IV) criteria

^f^Based on endorsing any of the five Patient Health Questionnaire (PHQ) alcohol items

^g^Assessed from the mean score of all 11 items from the Current State- Posttraumatic Growth Index – Short Form (C-PTGI-SF); ORs refer to a 1 point change in mean score

^h^Assessed from the mean score of 3 self-mastery items from Pearlin and Schooler’s Self - Mastery Scale; ORs refer to a 1 point change in mean score

All life stressors were significantly associated with problematic anger except for sexual assault. As the number of reported childhood traumatic experiences increased, so did the magnitude of the association with problematic anger, such that the strongest association was observed for those reporting 3 or more experiences [AOR = 2.70; 95% CI 2.43, 3.00; PAR%, 3.6] compared with those reporting no childhood trauma. A similar pattern was observed among those who reported financial problems or worries, with those reporting being “bothered a lot” having the strongest association [AOR = 1.94, 95% CI 1.82, 2.06; PAR%, 14.0] with problematic anger compared with those who were not bothered. Significant associations were observed for all psychological health/well-being factors. The strongest magnitude of association was among those with comorbid PTSD and MDD [AOR = 4.43; 95% CI 4.09, 4.80; PAR%, 12.0] and those with PTSD only [AOR = 3.52; 95% CI 3.28, 3.78; PAR%, 12.0]. Problem drinking was also associated with problematic anger [AOR = 1.46; 95% CI 1.38, 1.55; PAR%, 6.6]. With regard to mitigating factors, as mean scores for positive perspective and self-mastery increased, odds for problematic anger significantly decreased. Specifically, for each one-unit *increase* in mean score for positive perspective, there was an 28% decrease in odds for problematic anger (PAR%, 12.1 for a mean score increase from “moderate degree” to “a very great degree”). For every one-unit increase in mean score for self-mastery, there was a 16% decrease in odds for problematic anger (PAR%, 9.3 for a mean score increase from “neither agree or disagree” to “strongly agree”).

## Discussion

The present study documents that more than one in six responders to the 2016 Millennium Cohort Study survey reported levels of anger that are considered to be problematic based on the DAR-5 cutoff score of 12 points or higher. These findings provide an unprecedented examination of the scope of problematic anger in a robust sample of service members and veterans.

The present study also supports previous work that has found factors such as male gender, younger age, lower educational attainment, non-white race/ethnicity, and childhood trauma to be associated with anger [14, 44, 45]. Notably, in the case of non-white race/ethnicity, anger may be a reflection of longstanding social inequality or other forms of discrimination without the societally-sanctioned opportunity to express outrage [46]. While not the focus of the present study, these findings highlight the importance of considering differences in the way anger is understood within a diverse military community.

In addition to corroborating findings from other research, this study identified that being married, financial problems, and military-specific factors were significantly associated with higher odds for problematic anger, including serving on active duty, deployment with high levels of combat experience, and separation from military service. Importantly, these relationships remained significant after adjusting for baseline mental health disorders, including PTSD and MDD, suggesting that problematic anger appears to be more than simply an expression of PTSD and MDD. Instead, problematic anger appears to manifest in individuals with a constellation of factors reflecting personal background, aspects of the military experience, financial concerns, and mental health problems. Some of these factors may be temporary (e.g., financial concerns, mental health concerns), although the exact duration of predictors is beyond the scope of the present study.

Perhaps one benefit of identifying the significant and independent relationships between a range of predictors and problematic anger is that there is a multitude of potential points for targeting interventions. The PAR%s provide insight regarding which factors may be most influential in reducing problematic anger in this population. For example, by preventing or eliminating some of these modifiable or treatable conditions, such as financial problems, problem drinking, and PTSD, problematic anger may be reduced by 14.0, 6.6, and 12.0% respectively, assuming that these observed associations are causal and that elimination of these risk factors do not affect the distribution of other covariates. Conversely, increasing positive perspective and self-mastery (i.e., from mean score of 3 [“moderate agree” or “neither agree or disagree”] to 5 [“a very great degree” or “strongly agree”]) could lead to a reduction in problematic anger by 12.1 and 9.3%, respectively. While other ORs had a large magnitude, such as childhood trauma and MDD, the PAR% demonstrates that reductions in these factors would have a smaller effect (< 3% reduction) due to their lower prevalence in this population. These findings indicate that the effective prevention and treatment of modifiable conditions, such as problem drinking and PTSD, as well as the enhancement of positive perspective and self-mastery may reduce the prevalence of problematic anger.

Such interventions should be developed for military-specific application given the unique occupational context in which service members operate. Notably, the military occupational context trains individuals to maintain vigilance to threat and to consider confrontational strategies in the face of threat [47], which can potentially exacerbate anger. Not only should interventions take this context into account, but adaptation of existing interventions for the military context can be particularly beneficial since service members are more likely to engage in an intervention they regard as tailored for their needs [48].

Thus, developing interventions that target problematic anger in the military is critical given its high prevalence, distinction from other mental disorders, role in impeding effective PTSD treatment, and impact on vocational and interpersonal functioning. Results also suggest that directly targeting problematic anger may reduce the risk for interpersonal aggression and family violence, given research indicating that elevations on the DAR-5 conferred a 13 fold risk of aggression [Cowlishaw, Metcalf, Varker, Stone, Alkemade, Molyneaux, Gibbs, Block, Harms, MacDougall, Gallagher, Bryant, Lawrence-Wood, Kellett, O'Donnell and Forbes: Problematic anger prevalence and risk for aggression and suicidality in a post-disaster context, submitted]. The current findings further reinforced this elevated risk with over one in 10 respondents endorsing that when they get angry at someone, they wanted “to hit or clobber the person.” Direct targeting of anger also potentially opens the gateway to improved outcomes in co-occurring conditions such as military-related PTSD. It will be important for future research to distinguish between predictors of long-held problematic anger and recently established problematic anger in order to develop optimally tailored intervention strategies. Although there has been little empirical attention paid to the development and testing of direct interventions for problematic anger in military populations, approaches to the management and treatment of problematic anger in military populations have been recently developed and pilot tested in both the US [49] and Australia [47]. Implementing these kinds of specific interventions may also help mitigate the poorer treatment outcomes found with military veterans with PTSD relative to their civilian counterparts [50].

Besides clinical treatment for pathological responses, early intervention programs can also be developed for lower intensity expressions of anger. Early intervention strategies could be inserted into nontraditional contexts such as preparing financial counselors to reinforce military-specific anger management techniques with service members bothered by financial problems [48]. Early interventions could also include training counselors who focus on the pivotal process of transitioning from military to veteran status in targeted anger management. Such initiatives like the US Army’s Transition Assistance Program could integrate these techniques into their formal courses. Early interventions could also be adapted for military leaders, given the importance of leaders in establishing unit culture and influencing soldier health and wellbeing [51]. Results also highlight potential candidates for screening efforts and mental health treatments that are adapted to target anger as a primary goal.

The findings of the present study also point to another area of potential intervention, currently untapped in the conceptualization of anger responses and intervention. Specifically, findings indicate that individual endorsement of positive perspective and self-mastery is associated with reduced odds of problematic anger years later. These attitudes reflect the ability to use coping skills [52] that place challenges into perspective and that reflect the importance of finding meaning in one’s life and work. Skills related to these concepts could be strengthened in service members directly through training [52, 53], and indirectly through institutional messaging about the importance of positive perspective and self-mastery, and through leaders who set an example and reinforce these attitudes. By supporting these kinds of adaptive skills and perceptions, studies could examine whether the risk of problematic anger years later may be lowered.

While the present study examines factors associated with problematic anger over a 3–4 year follow-up period using a large sample, there are limitations. These data are correlational and there was no measure of DAR-5 on the baseline (2013) survey, which prevented the analysis of modeling the emergence of problematic anger or distinguishing between long-held and recently established problematic anger. In addition, self-reported survey data may be subject to recall and reporting biases. Finally, while the Millennium Cohort Study includes participants from all branches of service, active and Reserve components, and veterans, it may not necessarily be representative of the entire U.S. military or those who deploy. However, investigations of the Millennium Cohort Study have not demonstrated systematic sampling bias [28, 54].

## Conclusion

Results from this prospective study underscore the relatively high prevalence of problematic anger in a large military sample and the markers of risk and resilience that can potentially influence problematic anger over a period of time. By reducing the risk of problematic anger, individuals and organizations like the military may be able to benefit in terms of improved employee health, relationships with family, friends and coworkers, occupational functioning and reduced risks for aggression and violence.

## Data Availability

The data that support the findings of this study are not currently publicly available due institutional regulations protecting service member survey responses but are available from the corresponding author on reasonable request (may require data use agreements to be developed).

## Abbreviations

PTSD: Posttraumatic stress disorder
STAXI-2: State - Trait Anger Expression Inventory - 2
DAR-5: Dimension of Anger Reactions scale
PCL-C: PTSD Checklist-Civilian version
DSM-IV: Diagnostic and Statistical Manual of Mental Disorders 4th edition
MDD: Major depressive disorder
PTGI-SF: Posttraumatic Growth Inventory-Short Form
PAR%: Population attributable risk percent
AOR: Adjusted odds ratio
CI: Confidence interval
